# A278 ACCEPTABILITY, FEASIBILITY, AND IMPACT OF THE MYGUT DIGITAL HEALTH PLATFORM IN THE MONITORING AND MANAGEMENT OF INFLAMMATORY BOWEL DISEASE

**DOI:** 10.1093/jcag/gwad061.278

**Published:** 2024-02-14

**Authors:** J Zhen, M Simoneau, P Sharma, P Germain, J Marshall, W Afif, N Narula

**Affiliations:** University of Ottawa, Ottawa, ON, Canada; McGill University, Montreal, QC, Canada; McMaster University, Hamilton, ON, Canada; McGill University, Montreal, QC, Canada; McMaster University, Hamilton, ON, Canada; McGill University, Montreal, QC, Canada; McMaster University, Hamilton, ON, Canada

## Abstract

**Background:**

Inflammatory bowel disease (IBD) is a chronic gastrointestinal disorder that has been associated with depression, anxiety, and decreased quality of life. With new technological advancements, digital health monitoring platforms offer a promising avenue to integrate self-management strategies into the care of patients with IBD.

**Aims:**

This study aimed to investigate patients’ acceptability to implementing the MyGut IBD health monitoring platform into their care, evaluate whether its use leads to better quality of life/improved health outcomes, and determine the feasibility of its long-term use.

**Methods:**

We conducted a multi-centre, single-arm trial at McMaster University and McGill University, among their affiliated hospitals, from September 2020 to September 2023. Patients with IBD were recruited in gastroenterology clinics and asked to install the MyGut application onto their mobile devices. Various metrics such as willingness to use the application, patient satisfaction, symptom control/quality of life scores (measured through the short inflammatory bowel disease questionnaire (SIBDQ)), resource utilization, and feasibility statements were collected throughout the study. Patients exhibiting abnormal SIBDQ scores were identified for follow-up. After one year, patient outcome metrics were compared to baseline values.

**Results:**

Out of the 84 patients initially enrolled, 58 patients (69%) continued until study completion at one-year. At recruitment, all 84 patients (100%) were willing to use the MyGut application after a brief tutorial and 72.6% (61/84) had already been using technology for health-related purposes. There was a significant improvement in SIBDQ scores after one year of MyGut use (median = 57.5, IQR 50.25–62) when compared to baseline (median = 54.5, IQR 47.25–58.75) (p = 0.01). However, only 42.9% of patients (21/49) were willing to continue using the application after one year, a significant decrease compared to the 71.9% (41/57) who were willing to continue after two months (p = 0.002). No differences were observed in patient satisfaction measures (p = 0.42 to 1) or number of ER visits/hospitalizations (p = 0.78) before and after one year of MyGut use.

**Conclusions:**

Overall, IBD is a condition that often necessitates frequent medical follow-up and may benefit from proactive symptom management strategies. This study demonstrates that patients with IBD are very willing to integrate digital health monitoring platforms into their care and these may subsequently lead to improved symptom control and enhanced quality of life. Future studies should aim to investigate the subset of patients that would benefit most from these health interventions, and as illustrated, continued efforts must be made to optimize the acceptability/feasibility of its long-term use.

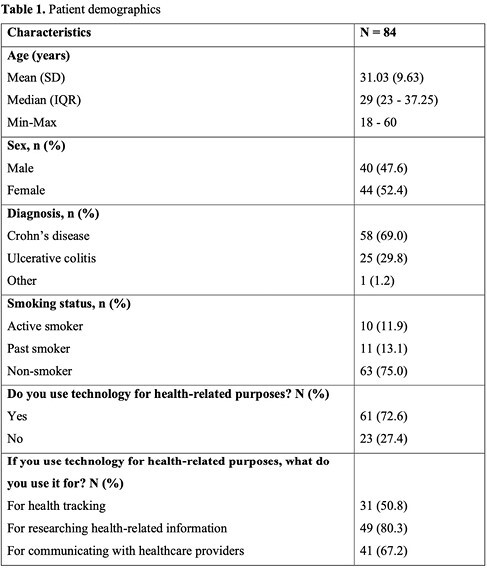

**Funding Agencies:**

CCC

